# How do German medical students perceive role models during clinical placements (“Famulatur”)? An empirical study

**DOI:** 10.1186/s12909-019-1624-9

**Published:** 2019-06-03

**Authors:** Oliver Keis, Achim Schneider, Felix Heindl, Markus Huber-Lang, Wolfgang Öchsner, Claudia Grab-Kroll

**Affiliations:** 1 Medical Faculty, Office of the Dean of Studies, University of Ulm, Albert Einstein Allee 7, 89081 Ulm, Germany; 2 Institute for Clinical and Experimental Trauma Immunology, Ulm University Medical Center, Helmholtzstr. 8/2, 89081 Ulm, Germany; 3 Center for Surgery / Department for Cardiac Anesthesiology, Ulm University Medical Center, Albert-Einstein-Allee 23, 89081 Ulm, Germany

**Keywords:** Role models, Clinical placements, Clinical clerkship, Qualities, Medical professions, Learning gains, Role modelling

## Abstract

**Background:**

Studies have demonstrated the importance of role models in medical education. Medical students in Germany participate in clinical placements (“Famulatur”) that last 4 months in total and represent the first real-world setting where students encounter possible role models in their clinical education. These placements are an extracurricular activity, however, and regarded as the “black box” of medical education. This study aimed to evaluate whether and how students experience role models during clinical placements, the qualities associated with potential role models and whether role model-related learning gains are relevant.

**Methods:**

We recruited 96 students (mean age: 23.83 years; 75% female) in their 5th to 9th semesters at the Faculty of Medicine at the University of Ulm, Germany, who were participating in a clinical placement between July and October 2015. Participants completed a questionnaire at the beginning of a 5-day working week to record sociodemographic and other information and another one at the end of the week to assess various aspects of their experiences. On each of the 5 days, they completed a structured questionnaire to record their perceived role models and self-assessed learning gains.

**Results:**

Role models and role modelling play an important role in clinical placements. The positive function of medical staff as role models predominated (88.4%) across all specialties. Junior doctors were the most frequently perceived role models (28.5%), followed by consultants (25.1%) and nursing staff (22.4%). The most commonly perceived positive quality was the interaction with students (16.5%), followed by team behaviour (13.6%), interaction with patients (13.6%) and professional expertise (13.4%). Students also had various kinds of learning gains such as knowledge or skills.

**Conclusions:**

Although these clinical placements are extracurricular activities in Germany and their content is not regulated, they are home to a relevant amount of role modelling. Students experience the various medical professions in different roles and in a range of tasks and interactions. Defining basic learning objectives could help to increase the relevance of these placements for the medical curriculum in Germany and transfer the associated learning gains from the hidden to the open curriculum.

**Electronic supplementary material:**

The online version of this article (10.1186/s12909-019-1624-9) contains supplementary material, which is available to authorized users.

## Background

Role models and role modelling play an important role in medical training but because of their often implicit nature [1, 2], less attention is paid to the related didactical approaches or teaching methods. As a rule, role modelling is not actively used in medical education but often takes place in the hidden curriculum of the clinical phases.

At the same time, a growing number of studies indicate the importance of role models in medical education. They play a decisive role in the development of professionalism [3], which is why students consider positive role models to be especially important in their learning environment [4]. Exemplary characteristics have been defined in particular as clinical expertise, patient care qualities, quality of teaching and human-personal qualities [1, 5]. Role models influence students in their career decisions [5], affect their choice of the clinical field for advanced medical training [6], and the professional behaviour of teachers has been identified as the most important factor influencing postgraduate education choices [7]. The relationship between positive and negative role models and career choices has been demonstrated in several fields, also outside medicine [8–11].

These insights are not only theoretical but are also directly relevant for practice: if perceived role models influence students’ choice of specialty for their further training, we must consider the associated consequences for disciplines that tend to experience recruitment difficulties, e.g. general practice. Accordingly, suitable role models and best practice examples might be an effective way for specialties to attract new talent independent of other factors, e.g. salary.

Against this background, when developing role models among the academic staff of medical specialties it is recommended that the impact of humanistic and professional characteristics should be emphasised [12]. All in all, role modelling is an important teaching tool for “passing on the knowledge, skills and values of the medical profession […].” [13].

The 6 year German medical curriculum consists of two parts: a preclinical section lasting 2 years is followed by a clinical section of 4 years duration. The final year of the clinical section, known as practical year, is a full-time clinical practical training of 48 weeks. In the clinical section of their education, German medical students first experience practising doctors and medical staff in the “Famulatur” - mandatory clinical placements (that are also referred to as medical/clinical clerkships or clinical electives) that students have to complete after the preclinical section and before the practical year. The “Famulatur” lasts 4 months in total and aims to familiarise students with clinical patient care in facilities providing in- and outpatient services under medical supervision [14]. Beyond that, there are no clear guidelines concerning rotations or responsibilities during these placements.

Although the placements are mandatory and represent a considerable portion of the clinical section, there are no curricular standards for their learning content and learning objectives and, paradoxically, they take place outside the curricula of medical education. Thus, during these 4 months of students’ contact with clinical practice the supervising doctors and medical staff in the educational institutions have a special responsibility and importance because their behaviour can decisively influence the students’ view of the respective specialty. Additionally, the experience of clinical practice in clinical placements has been identified as a factor that may encourage students to opt for a career in the corresponding specialty [15, 16].

Despite growing research on clerkship education [17], few studies have been performed on the effects of role models on medical students during these clinical placements. The “Famulatur” continues to rather be a “black box” within medical education in Germany and the associated learning content can best be described as part of a hidden curriculum. Therefore, the goal of the present study was to answer the following questions:

(1) To what measurable extent do medical students perceive role models during a 5 day-week of a clinical placement and in which groups of people do they experience role models?

(2) Which qualities – in the following “qualities” refers to behaviours and characteristics that are relevant for medical training – do students notice in the perceived role models and which groups of people are associated with which qualities?

(3) Which groups of people affect how students rate the attractiveness of a specialty?

(4) Do role modelling and role model-based learning play a relevant role in the clinical placements?

By answering these questions, we aimed to gather more information on the clinical placements and to contribute to the growing pool of studies on the topic of role models in medicine.

## Methods

### Participants

In July 2015 we wrote to all students in the 5th to 9th semester (*N* = 722) at the Faculty of Medicine of the University of Ulm, Germany, and invited them to participate in the study. To be able to participate, the students had to be starting a clinical placement in the summer break between July and October 2015 but were free to choose in which week of their clinical placement they wanted to undertake the study.

Under these conditions, we were able to recruit 96 participants into the study (response rate: 13%; mean age: 23.8 years; 75% women). At the time of the study, these students started a clinical placement in the following 13 specialties: internal medicine (*n* = 24), surgery (*n* = 13), anaesthesiology (n = 13), general practice (*n* = 12), psychiatry (*n* = 6), gynaecology (*n* = 5), neurology (n = 5), radiology (*n* = 4), urology (n = 2), paediatrics (n = 2), orthopaedics (n = 2), dermatology (n = 1) and otorhinolaryngology (n = 1). On average, study participants had completed 7.7 weeks (SD 4.8) of their clinical placements at the time of the study.

Participants provided written informed consent after the aim of the study had been explained to them and they were assured that their data would remain anonymous for data analysis. After submitting the completed questionnaires (one at the beginning of a 5-day working week during the placement, one at the end of the week and one in the form of a 5-day study log), study participants received a compensation of 30 euros. Based on the guidelines of the local Independent Ethics Committee of Ulm University no specific ethical approval was required to perform this study.

### Research topic

After a preclinical section lasting 2 years, medical students in Germany enter the clinical section of their medical studies. The clinical section lasts 4 years in total and according to the German Medical Licensing Regulations for Physicians (“Approbationsordnung für Ärzte”) students must complete the “Famulatur” in this section - clinical placements lasting 4 months in total [14]. This 4-month clinical phase must include 1 month in an outpatient facility, 2 months in a hospital or inpatient rehabilitation facility and 1 month in a general practice facility; each part can be divided into a maximum of 5 segments, each of which must last at least 14 calendar days, and must be completed during the lecture-free time after the preclinical section and before the practical year. The students work full time and should thereby actively participate in patient care, supervised by medical attendants. Besides these requirements, students organise these placements by themselves and there are no guidelines concerning students’ responsibilities or choice of facility during these placements.

In contrast to the other practical sections of the degree such as the practical year, which is a full-time clinical practical training of 48 weeks in the final year of the clinical phase and is part of the curriculum, the clinical placements are mandatory but extracurricular and have no explicit learning objectives.

### Data collection

In addition to asking about sociodemographic (age, sex) and other variables, the initial questionnaire before the start of a 5-day working week during the clinical placement asked about previous experience of clinical placements (number of weeks) and the chosen specialty.

In the concluding questionnaire at the end of a 5-day week, the students had to state among other things whether their experiences had increased the attractiveness of the chosen specialty as a personal career goal (response options: 1 = strongly disagree to 5 = strongly agree) or decreased it (response options: 1 = strongly disagree to 5 = strongly agree).

Besides these two questionnaires, on every day of the 5 consecutive work days being studied the study participants used a structured questionnaire to record their perception of role models and their perceived learning gains; each day was divided into “morning” and “afternoon” and participants were asked to complete the respective section at mid-day and in the evening. For both parts of the day the students had to state whether they had perceived role models (yes/no response), making a total of 10 opportunities to perceive role models (twice a day for 5 days). If students perceived role models they were asked to state whether the role models were positive or negative and in which of the following 5 groups of people they were perceived: junior doctor/ward doctor, consultant/senior consultant, nursing staff, other team member or other student (multiple responses were allowed). For each group of people, the study participants then had to indicate if they perceived any of the following eight qualities as positive or negative: professional expertise, team behaviour, interaction with patients, interaction with relatives, quality of teaching, interaction with students, human-personal characteristics and being well structured. Thus, for each quality the students could make a total of 50 statements (5 days each with 2 parts and 5 groups of people).

In addition to providing information on the perceived role models, the students had to specify in which domain they perceived learning gains for the respective part of the day. The learning domains included knowledge/theory, proficiency/skills, social competence in the team, patient presentation, work processes and documentation (response options: 1 = not at all to 5 = to a very great extent). The students then stated the extent to which they believed their learning gains were related to the perceived role models (response options: 1 = not at all to 5 = to a very great extent) (see Additional file 1 for questionnaires).

The completed questionnaires were entered into a file mask by two previously trained assistants who were otherwise not involved in the study.

### Data analysis and statistics

After checking the plausibility of the data, we calculated sums and means of the data in the 5-day study logs. This included the total number of registered perceptions of role models, total number of positive and negative role models in the different groups of people and the total number of qualities both across the 5 groups of people and separately for each group. For the data on self-perceived learning gains and the associated estimate of the respective importance of role modelling, we calculated means across the various time points in the study log.

Differences between the number of perceived role models in the morning and afternoon were analysed with a Wilcoxon signed-rank test because data did not show a normal distribution. Associations between perceptions of role models and students’ ratings were analysed by Spearman’s rank correlation coefficient (*ρ*).

Data analysis was performed with IBM SPSS Statistics for Windows (Version 25.0.0.0).

## Results

The number of perceived role models was slightly different in the morning and afternoon (*M*_morning_ = 4.75, *M*_afternoon_ = 4.49, *p* = 0.01). However, because this difference is of minor importance and not relevant for the study aims it will not be discussed below.

### Quantitative perception of role models during the clinical placement

Role models were perceived in a mean of 9.24 (SD 1.23) of 10 potential opportunities. Junior doctors represented the most common role models, closely followed by consultants and nursing staff; other students were also occasionally experienced as role models (see Table 1). Across all groups of people, most role models were positive (88.4%) but some were negative (11.6%). The percentage of role models perceived as being positive or negative was similar within each group (see Table 1).

**Table 1:** Relative (%) and absolute (mean, SD) number of perceived role models (positive and negative) in the morning and afternoon of a 5-day week of a clinical placement (= maximum of 10 possibilities per group of people) and relative frequency of positive and negative perceived role models (%) in each group of people

Group of people	Perceived role models		Positive (in %)	Negative (in %)
	% of total	Mean (SD)		
Junior doctors/Ward doctors	28.5	7.19 (3.00)	6.49 (90.3)	0.7 (9.7)
Consultant/Senior consultants	25.1	6.35 (2.68)	5.57 (87.7)	0.78 (12.3)
Nursing staff	22.4	5.66 (3.12)	4.9 (86.6)	0.76 (13.4)
Other team members	9.4	2.37 (2.56)	2.06 (86.9)	0.31 (13.1)
Other students	14.6	3.69 (3.18)	3.31 (89.7)	0.38 (10.3)
Total	100	25.26 (8.58)	22.33 (88.4)	2.93 (11.6)

### Frequency of qualities

#### Qualities perceived during the clinical placement

Interaction with students was a particularly relevant quality. The participants most frequently experienced exemplary behaviour related to the quality “interaction with students”, followed by “interaction with patients” and “team behaviour” (Table 2). The category “interaction with students” was also the most frequently noted negative quality, followed by human-personal characteristics and team behaviour. Perception of the quality “interaction with students” as positive also seemed to increase with growing experience of clinical placements: we found a significant association between clinical placement experience (in weeks) and frequency of mentions of this quality (*ρ* = 0.337, *p* = 0.001; data not shown).

**Table 2:** Mean (SD) number of times each quality was perceived as positive or negative and relative frequency (%) across the 5 groups of people in the morning and afternoon of a 5-day week of a clinical placement (= maximum of 50 possibilities per quality)

Qualities	Perceived as positive		Perceived as negative		Total perceived	
	Mean (SD)	%	Mean (SD)	%	Mean (SD)	%
Professional expertise	9.93 (6.18)	13.4	0.59 (1.22)	0.8	10.52 (6.28)	14.2
Team behaviour	10.11 (7.11)	13.6	1.01 (1.80)	1.4	11.13 (7.41)	15.0
Interaction with patients	10.06 (6.52)	13.6	0.54 (0.87)	0.7	10.6 (6.69)	14.3
Interaction with relatives	3.19 (3.59)	4.3	0.23 (0.59)	0.3	3.42 (3.69)	4.6
Quality of teaching	7.8 (5.06)	10.5	0.83 (2.15)	1.1	8.64 (5.49)	11.7
Interaction with students	12.23 (7.58)	16.5	1.24 (2.29)	1.7	13.47 (8.36)	18.2
Human-personal characteristics	9.72 (6.21)	13.1	0.96 (1.57)	1.3	10.68 (6.42)	14.4
Being well structured	4.9 (4.28)	6.6	0.76 (1.47)	1.0	5.66 (4.46)	7.6
Total	67.94 (34.71)	91.7	6.17 (8.67)	8.3	74.10 (36.12)	100

The interaction with relatives was the least frequently mentioned quality, both as a positive and negative quality (Table 2).

#### Perceived positive and negative qualities in the various groups of people

**Fig. 1:**
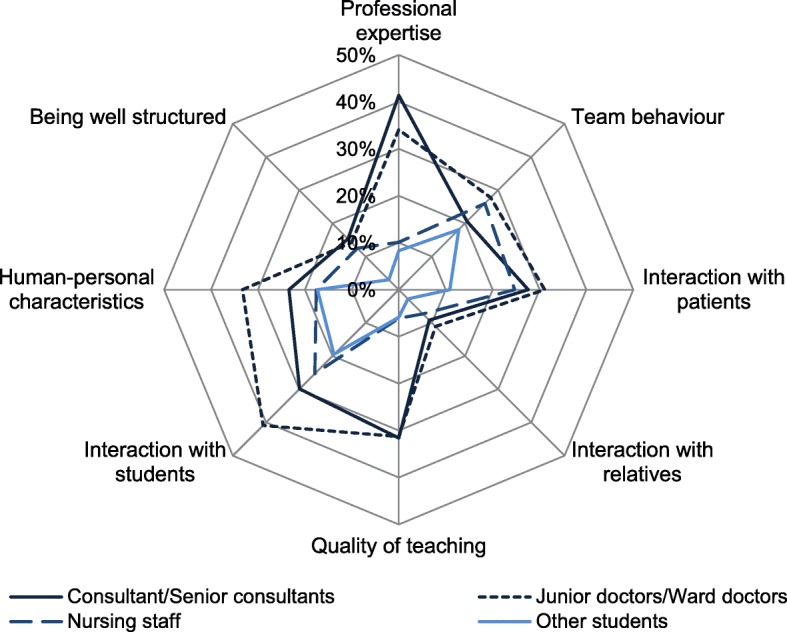
Frequency of perception of positive role model qualities in the different groups of people (percent of a maximum of 10 possible encounters)

We found differences in the positive qualities perceived by the students in the various groups of people. Each group of people was characterised by particular features, as shown in the spider chart in Fig. 1.

In the group of medical professionals most often perceived as role models, the junior doctors, their interaction with students was the most common positive experience, followed by professional expertise and human-personal characteristics. Consultants were most frequently characterised by their professional expertise, followed by the quality of their teaching and interaction with students. Among the nursing staff, the third most frequently perceived group of medical professionals, the participants most frequently noted their behaviour in the team and their interaction with students and patients (Fig. 1).

**Fig. 2:**
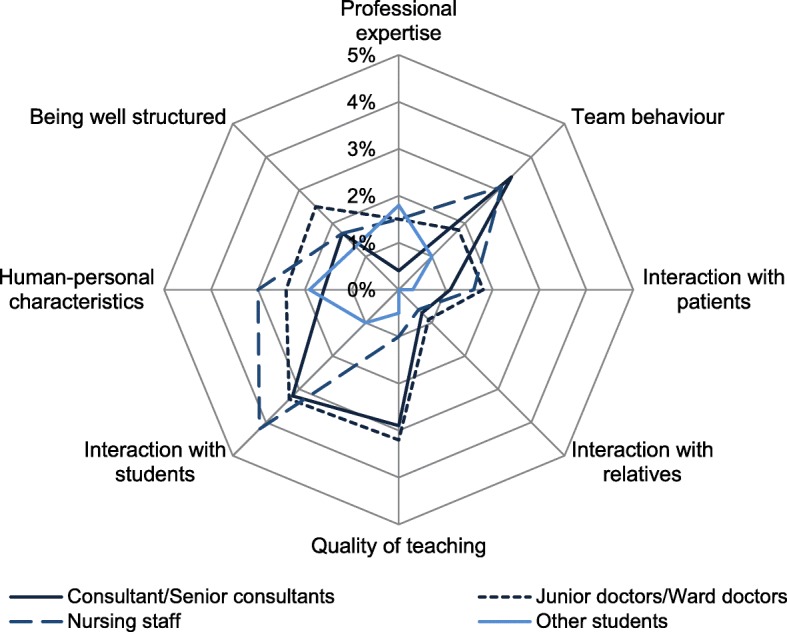
Frequency of perception of negative role model qualities in the different groups of people (percent of a maximum of 10 possible encounters)

Negative qualities were much less common than positive ones. The most common negative quality in role models was the interaction of nursing staff with students. Consultants’ behaviour in the team and the interaction of junior doctors with students were the second and third most frequently named negative qualities in role models (Fig. 2).

The interaction with relatives was only rarely perceived as a positive or negative quality across all groups of people.

#### Correlation between role models and the attractiveness of a specialty

The groups of medical professionals most often perceived as role models by students in the 5 days of the clinical placement, the consultants and junior doctors, were also associated with the students’ ratings of the attractiveness of the respective specialty.

In the group of consultants, we found a significant association between the perception of the consultant as a positive role model and the students’ rating that the clinical placement increased the attractiveness of the specialty (*ρ* = 0.243, *p* = 0.017).

In the group of junior doctors/ward doctors, we found a significant association between the perception of the junior doctor as a negative role model and the students’ rating that the clinical placement decreased the attractiveness of the specialty (*ρ* = 0.262, *p* = 0.010).

In the other groups perceived as role models during the clinical attachment, we found no significant associations between the perception of role models and the assessment of attractiveness of a specialty.

#### Role model-related learning gains during the clinical placement

The study participants stated that role modelling or role model-related learning gains played a key role during the 5 days of the clinical placement (mean [SD]: 3.60 [0.648]). Self-assessed learning gains thereby correlated with the perception of role models. Thus, students who experienced more positive role models also stated that they had greater learning gains in various learning domains (see Table 3).

**Table 3:** Relationships between self-assessed learning gains and perceived role models shown as correlation coefficients (Spearman’s rho)

	Positive perceptions of role models			Negative perceptions of role models		
	Consultant	Junior doctor	Nursing staff	Consultant	Junior doctor	Nursing staff
Knowledge/theory	0.43^***^	0.12	0.02	−0.32^**^	− 0.14	0.11
Proficiency/skills	0.43^***^	0.14	0.10	−0.28^**^	−0.18	0.13
Social competence in the team	0.29^**^	0.20	0.24^*^	−0.31^**^	−0.18	0.12
Patient presentation	0.11	0.25^*^	0.09	−0.17	0.02	0.19
Work processes	0.38^***^	0.25^*^	0.30^**^	−0.26^*^	− 0.14	0.21^*^
Documentation	0.33^**^	0.22^*^	0.27^*^	−0.17	−0.10	0.05

^*^p < 0.05, ^**^p < 0.01, ^***^p < 0.001

With respect to the professional groups, the perception of consultants as positive or negative role models was particularly important for learning. If the consultant was more frequently perceived as a positive role model, the students stated that they had greater learning gains in the domains knowledge/theory, proficiency/skills, social competence in the team, work processes and documentation. In contrast, perception of the consultant as a negative role model was significantly associated with less learning gains in the domains knowledge/theory, proficiency/skills, social competence in the team and work processes.

We also found a significant association between the frequency with which junior doctors were perceived as positive role models and the learning related to patient presentation, work processes and documentation.

Perception of the nursing staff as a positive role model was significantly associated with learning gains in the domains social competence in the team, work processes and documentation. In terms of perception as a negative role model we found a significant association with work processes.

## Discussion

As our study shows, role models and role modelling are an important aspect of clinical placements. During their placement, students perceived role models differently in the various groups of people and were well able to differentiate between positive and negative role models. Studies of medical students in other contexts also found that they were able to discern professional or unprofessional behaviours [18, 19].

The most frequently perceived positive quality was the interaction with students, indicating that the students had a very good sense of how people are dealing with them. This perception increased with growing experience of clinical placements. Professional expertise, team behaviour and interaction with patients were also frequently perceived as positive qualities. These findings are consistent with other studies emphasising professional expertise or patient care qualities as exemplary characteristics of role models [1, 5].

In contrast, the interaction with relatives seemed to play a minor role because study participants seldom mentioned this quality. This could be attributed to the sensitivity of such situations, leading to an exclusion of students. Although reasons for this finding remain unclear, it should be possible even for students that enter a clinical placement for the first time to participate in selected conversations between medical staff and relatives. It might be that the responsible parties at training facilities should better explain such qualities to medical staff.

The finding that junior doctors were the most frequently perceived role model is not too surprising, given that they are usually the direct supervisors of the students. A review in the field of surgery also stresses the opportunity for residents/junior doctors to act as mentors or role models since they have the greatest amount of exposure to the students [20]. The next most frequently perceived role models were consultants, but nursing staff were also an important reference group for the students.

Our findings also clearly show that specific qualities were perceived in each group of medical professionals. Thus, in consultants/senior consultants the students most frequently perceived professional expertise as exemplary, in nursing staff the interaction with students and patients and the team behaviour and in junior doctors/ward doctors also the interaction with students. These findings highlight the respective relevance of all these groups during clinical placements and also the importance of allowing students in clinical placements to have sufficient contact with the various groups of medical professionals. Of relevance in this context is that the role model behaviour of clinical trainers can be trained or improved [21].

The relationships we found between the experience of positive or negative role models and the rating of the attractiveness of the specialty chosen for the clinical placement may be particularly relevant when it comes to attracting potential future doctors or junior staff to a specialty. The students’ perception of consultants and junior doctors as role models was associated with their rating of the attractiveness of a specialty. Making these two groups aware of their respective effect on students, whether during clinical placements or in other clinical phases of medical education, could be a suitable, cost-effective means to improve the situation in specialties with a current or predicted shortage of professional staff.

The study results are also interesting with regard to students’ self-assessed learning gains during the clinical placement. We found a relationship between the perception of role models and learning in that students who perceived more positive role models noted more learning gains in the various domains. Consultants again played a special role in this context: if the consultant was frequently perceived as a positive or negative role model, the student noted higher or lower learning gains, respectively, in the various domains.

The finding that consultants were not the most frequently perceived role models but played an important role in various contexts may indicate the impact of the hierarchical structure for role modelling.

Our results show that although clinical placements in German medical education are extracurricular and their content is therefore not regulated in any way (only by formal requirements), a relevant amount of role modelling and learning take place in them. Many of the aspects conveyed by role models during clinical placements can be attributed to the informal, hidden curriculum [22, 23]. Considering other findings highlighting the importance of doctor role modelling for professional or character development [24] it seems short-sighted to underestimate the relevance of clinical placements in the continued development of the medical education curriculum and to place no respective value on them or to leave their added educational value in the hidden curriculum.

The “Masterplan Medizinstudium 2020” (Master Plan Medical Education 2020, [25]) and the associated integration of essential parts of the “National Competence-based Catalogue of Learning Objectives for Medicine” (NKLM - Nationaler Kompetenzbasierter Lernzielkatalog Medizin; [26]) will realign medical education in Germany. Many of the aspects described in the NKLM with respect to the “Role of the doctor”, which build on the role of the CanMEDS (Canadian Medical Education Directives for Specialists) framework [27], are already addressed by the clinical placement. Thus, doctors and other medical professionals not only act as team members but also as professional agents, communicators, medical experts and teachers. The realignment of medical education should also increase the importance of cooperation within interprofessional teams in the future. In order for this requirement to be more firmly anchored in the professional self-image of the various medical professions, the training provider/educational planner must promote a positive attitude towards interprofessional collaboration. Clinical placements could also contribute to this goal.

A limitation of our study is that it did not compare role models and role modelling in various specialties because the sample size for the individual specialties was too small. Further research is needed on this topic. As an additional research approach, we suggest performing a longitudinal study to evaluate the extent to which students’ experiences in clinical placements influence the choice of the future professional field. For example, is there a lasting association between the positive role models experienced during a clinical placement in a specialty and a related preference for that specialty when determining the later career goals/professional field? If so, this would be further proof of the importance of role models in medical education. Another weakness of our study is the single institution focus and that students were presented with closed choices both in terms of qualities and learning outcomes. To gain further insights on the impacts of role models qualitative research approaches could be useful.

## Conclusions

Our findings indicate that it may be useful for curriculum developers in Germany to place greater emphasis on the mandatory, extracurricular clinical placement. Even though the content of this 4-month part of students’ clinical training is not (yet) regulated, a relevant amount of informal learning or role modelling takes place in it. During the clinical placements students experience medical staff in different roles, as defined in the CanMEDS framework and NKLM, and also interprofessional teamwork.

To anchor these elements more strongly in the curriculum and thus increase their value, it may be advisable to improve the structure of the clinical placement. One such possibility may be for medical faculties to define the competences that should be promoted during this part of students’ education. These competences include basic examination techniques, basic rules for communication between doctors and patients, the correct use of medical terminology in medical treatment teams and analysis of doctors’ role. With these objectives at hand the role modelling identified in the present study, which is often implicit learning, could be linked with explicitly formulated learning objectives and thus at least partially transfer the learning from the hidden to the open curriculum. Not only the students but also all the medical staff could benefit from the resulting increase in the value of both aspects in this phase of the students’ education.

## Additional file

Additional file 1: Study questionnaires. (PDF 581 kb)

## Data Availability

The datasets obtained during and/or analysed during the current study are available from the corresponding author on reasonable request.

## Abbreviations

CanMEDS: Canadian Medical Education Directives for Specialists
NKLM: Nationaler Kompetenzbasierter Lernzielkatalog Medizin (National Competence-based Catalogue of Learning Objectives for Medicine)

## References

[1] Jochemsen-van der Leeuw HGA, van Dijk N, van Etten-Jamludin FS, Wieringa-de Waard M (2013). Attributes of the clinical trainer as a role model: a systematic review. Acad Med.

[2] Lunenberg M, Korthagen F, Swennen A (2007). The teacher educator as a role model. Teach Teach Educ.

[3] Kenny NP, Mann KV, MacLeod H (2003). Role modeling in physicians’ professional formation: reconsidering an essential but untapped educational strategy. Acad Med.

[4] Byszewski A, Hendelman W, McGuinty C, Moineau G (2012). Wanted: role models — medical students' perceptions of professionalism. BMC Med Educ.

[5] Passi V, Johnson S, Peile E, Wright S, Hafferty F, Johnson N (2013). Doctor role modelling in medical education. The BEME collaboration guide no 27. Med Tech.

[6] Wright S, Wong A, Newill C (1997). The impact of role models on medical students. J Gen Intern Med.

[7] Stahn B, Harendza S. Role models play the greatest role – a qualitative study on reasons for choosing postgraduate training at a university hospital. GMS Z Med Ausbild. 2014;31(4). 10.3205/zma000937 PMC4259064 25489345

[8] Mutha S, Takayama JI, O’Neil EH (1997). Insights into medical students’ career choices based on third-and fourth-year students’ focus-group discussions. Acad Med.

[9] Quimby JL, Santis AM (2006). The influence of role models on women’s career choices. Career Dev Q.

[10] Perrone KM, Zanardelli G, Worthington EL, Chartrand JM (2002). Role model influence on the career decidedness of college students. Coll Stud J.

[11] Buunk AP, Peiro JM, Griffioen C (2007). A positive role model may stimulate career-oriented behavior. J Appl Soc Psychol.

[12] Bahman-Bijari B, Zare M, Haghdoost AA, Bazrafshan A, Beigzadeh A, Esmaili M (2016). Factors associated with students’ perceptions of role modelling. Int J Med Educ.

[13] Cruess SR, Cruess RL, Steinert Y (2008). Role modelling – making the most of a powerful teaching strategy. BMJ..

[14] ÄAppO. Approbationsordnung für Ärzte vom 27. Juni 2002 (BGBl. I S. 2405), die durch Artikel 5 des Gesetzes vom 17. Juli 2017 (BGBl. I S. 2581) geändert worden ist. Retrieved from: https://www.gesetze-im-internet.de/_appro_2002/BJNR240500002.html. Accessed 29 May 2019.

[15] Maiorova T, Stevens F, Scherpbier A, Van der Zee J (2008). The impact of clerkships on students' specialty preferences: what do graduates learn for their profession?. Med Educ.

[16] Mihalynuk T, Leung G, Fraser J, Bates J, Snadden D (2006). Free choice and career choice: clerkship electives in medical education. Med Educ.

[17] Dornan T, Tan N, Boshuizen H, Gick R, Isba R, Mann K, Scherpbier A, Spencer J, Timmins E (2014). How and what do medical students learn in clerkships? Experience based learning (ExBL). Adv Health Sci Educ.

[18] Curry SE, Cortland CI, Graham M (2011). Role-modelling in the operating room: medical student observations of exemplary behaviour. Med Educ.

[19] Reddy ST, Farnan JM, Yoon JD, Leo T, Upadhyay GA, Humphrey HJ, Arora VM (2007). Third-year medical students' participation in and perceptions of unprofessional behaviors. Acad Med.

[20] Healy NA, Cantillon P, Malone C, Kerin MJ (2012). Role models and mentors in surgery. Am J Surg.

[21] Jochemsen-van der Leeuw HGA, Wieringa-de Waard M, Van Dijk N (2015). Feedback on role model behaviour: effective for clinical trainers?. Perspect Med Educ.

[22] Hafferty FW, Franks R (1994). The hidden curriculum, ethics teaching, and the structure of medical education. Acad Med.

[23] Stern DT (1998). In search of the informal curriculum: when and where professional values are taught. Acad Med.

[24] Passi V, Johnson N (2016). The impact of positive doctor role modeling. Med Teach.

[25] BMBF (2017). Masterplan Medizinstudium 2020. Retrieved from: https://www.bmbf.de/files/2017-03-31_Masterplan%20Beschlusstext.pdf. Accessed 29 May 2019.

[26] MFT Medizinischer Fakultätentag der Bundesrepublik Deutschland e. V. (2015) Nationaler Kompetenzbasierter Lernzielkatalog Medizin. Retrieved from: http://www.nklm.de/files/nklm_final_2015-07-03.pdf. Accessed 29 May 2019.

[27] Frank JR, Snell L, Sherbino J, editors. CanMEDS 2015 Physician Competency Framework. Ottawa: Royal College of Physicians and Surgeons of Canada; 2015.

