# Supplementary material for: How do German medical students perceive role models during clinical placements (“Famulatur”)? An empirical study
Source: BMC Med Educ. 2019 Jun 3;19:184. doi: 10.1186/s12909-019-1624-9 (PMC6547468; doi:10.1186/s12909-019-1624-9)
Supplement: Supplementary file 1 — Study questionnaires. (PDF 581 kb) [file 12909_2019_1624_MOESM1_ESM.pdf]

Clinical Placement Study: Initial Questionnaire

1. Please state your age: ……………………………………

2. Please state your sex: ◻ male ◻ female

3. How many weeks of your clinical placement(s) have you completed in total (current and previous)? ....

4. Have you completed previous vocational training? ◻ yes ◻ no

   If yes, what kind? …...

5. In which specialty are you currently performing a clinical placement? …...

6. Which ‘provider’ did you choose for the clinical placement?

   ◻ University hospital ◻ Municipal hospital ◻ Other provider: …..

7. Why did you choose this particular clinical placement? (multiple answers possible)

   ◻ Personal recommendation (e.g. from people in a clinical placement or other people)
   ◻ Personal contacts (e.g. with staff at the clinical placement site)
   ◻ General good reputation of the clinical placement site
   ◻ Proximity to home
   ◻ Payment
   ◻ The chosen location fits well with your own plans (timing, logistics, etc.)
   ◻ To gain insights into unfamiliar specialty
   ◻ To deepen existing knowledge
   ◻ To test possible career decisions
   ◻ Other reasons, namely: …...

8. How attractive for you as a personal career goal is the specialty you chose for the clinical placement?

Not at all attractive	Not very attractive	Neither attractive nor unattractive	Moderately attractive	Very attractive
◻	◻	◻	◻	◻

9. Which of the following factors play a role in this rating and to what extent?

	Not at all				To a great extent
▪ Work-life balance	◻	◻	◻	◻	◻
▪ Family friendliness	◻	◻	◻	◻	◻
▪ Patient contact	◻	◻	◻	◻	◻
▪ Personal enthusiasm	◻	◻	◻	◻	◻
▪ Role models and people you know who come from this specialty or are close to it	◻	◻	◻	◻	◻
▪ Social recognition, social status of the specialty	◻	◻	◻	◻	◻
▪ Importance for own education	◻	◻	◻	◻	◻
▪ Importance for own career	◻	◻	◻	◻	◻

Record of Learning

1st day - morning

1. To what extent did you have a learning gain in the following areas **this morning**?

	Not at all				To a very great extent
◦ Increase in knowledge (theory)	◻	◻	◻	◻	◻
◦ Increase in skills	◻	◻	◻	◻	◻
◦ Medical history taking	◻	◻	◻	◻	◻
◦ Other conversational skills with patients	◻	◻	◻	◻	◻
◦ Social competence in the team	◻	◻	◻	◻	◻
◦ Organisational knowledge	◻	◻	◻	◻	◻
◦ Work processes	◻	◻	◻	◻	◻
◦ Hygiene	◻	◻	◻	◻	◻
◦ Documentation, written tasks	◻	◻	◻	◻	◻
◦ Patient presentation	◻	◻	◻	◻	◻

2. To what extent was the overall learning gain attributable to 'observational learning, learning through or from role models'?

Not at all	Not very	Somewhat attributable, somewhat not	To a great extent	To a very great extent
◻	◻	◻	◻	◻

3. Did you experience or perceive role models **this morning** (positive or negative)? ◻ yes ◻ no

Junior doctors/ward doctors ◻ negative ◻ positive ◻ not at all

In which respect? (multiple answers possible)

◻ Professional expertise
◻ Team behaviour
◻ Interaction with patients
◻ Interaction with relatives
◻ Quality of teaching
◻ Interaction with students
◻ Human-personal characteristics (self-criticism, openness, patience, honesty)
◻ Being well structured

Consultants/Senior consultants ◻ negative ◻ positive ◻ not at all

In which respect? (multiple answers possible)

◻ Professional expertise
◻ Team behaviour
◻ Interaction with patients
◻ Interaction with relatives
◻ Quality of teaching
◻ Interaction with students
◻ Human-personal characteristics (self-criticism, openness, patience, honesty)
◻ Being well structured

**Nursing staff**	□ negative	□ positive	□ not at all

In which respect? (multiple answers possible)

□ Professional expertise	□ Team behaviour
□ Interaction with patients	□ Interaction with relatives
□ Quality of teaching	□ Interaction with students
□ Human-personal characteristics (self-criticism, openness, patience, honesty)	□ Being well structured

**Other team members**	□ negative	□ positive	□ not at all

In which respect? (multiple answers possible)

□ Professional expertise	□ Team behaviour
□ Interaction with patients	□ Interaction with relatives
□ Quality of teaching	□ Interaction with students
□ Human-personal characteristics (self-criticism, openness, patience, honesty)	□ Being well structured

**Other students**	□ negative	□ positive	□ not at all

In which respect? (multiple answers possible)

□ Professional expertise	□ Team behaviour
□ Interaction with patients	□ Interaction with relatives
□ Quality of teaching	□ Interaction with students
□ Human-personal characteristics (self-criticism, openness, patience, honesty)	□ Being well structured

**Others: ....**	□ negative	□ positive	□ not at all

In which respect? (multiple answers possible)

□ Professional expertise	□ Team behaviour
□ Interaction with patients	□ Interaction with relatives
□ Quality of teaching	□ Interaction with students
□ Human-personal characteristics (self-criticism, openness, patience, honesty)	□ Being well structured

Record of Learning

1st day - afternoon

1. To what extent did you have a learning gain in the following areas **this afternoon**?

	Not at all				To a very great extent
◦ Increase in knowledge (theory)	◻	◻	◻	◻	◻
◦ Increase in skills	◻	◻	◻	◻	◻
◦ Medical history taking	◻	◻	◻	◻	◻
◦ Other conversational skills with patients	◻	◻	◻	◻	◻
◦ Social competence in the team	◻	◻	◻	◻	◻
◦ Organisational knowledge	◻	◻	◻	◻	◻
◦ Work processes	◻	◻	◻	◻	◻
◦ Hygiene	◻	◻	◻	◻	◻
◦ Documentation, written tasks	◻	◻	◻	◻	◻
◦ Patient presentation	◻	◻	◻	◻	◻

2. To what extent was the overall learning gain attributable to 'observational learning, learning through or from role models'?

Not at all	Not very	Somewhat attributable, somewhat not	To a great extent	To a very great extent
◻	◻	◻	◻	◻

3. Did you experience or perceive role models **this afternoon** (positive or negative)? ◻ yes ◻ no

Junior doctors/ward doctors ◻ negative ◻ positive ◻ not at all

In which respect? (multiple answers possible)

◻ Professional expertise ◻ Team behaviour
◻ Interaction with patients ◻ Interaction with relatives
◻ Quality of teaching ◻ Interaction with students
◻ Human-personal characteristics (self-criticism, openness, patience, honesty) ◻ Being well structured

Consultants/Senior consultants ◻ negative ◻ positive ◻ not at all

In which respect? (multiple answers possible)

◻ Professional expertise ◻ Team behaviour
◻ Interaction with patients ◻ Interaction with relatives
◻ Quality of teaching ◻ Interaction with students
◻ Human-personal characteristics (self-criticism, openness, patience, honesty) ◻ Being well structured

**Nursing staff**	◻ negative	◻ positive	◻ not at all
In which respect? (multiple answers possible)	◻ Professional expertise	◻ Team behaviour	
	◻ Interaction with patients	◻ Interaction with relatives	
	◻ Quality of teaching	◻ Interaction with students	
	◻ Human-personal characteristics (self-criticism, openness, patience, honesty)	◻ Being well structured	
**Other team members**	◻ negative	◻ positive	◻ not at all
In which respect? (multiple answers possible)	◻ Professional expertise	◻ Team behaviour	
	◻ Interaction with patients	◻ Interaction with relatives	
	◻ Quality of teaching	◻ Interaction with students	
	◻ Human-personal characteristics (self-criticism, openness, patience, honesty)	◻ Being well structured	
**Other students**	◻ negative	◻ positive	◻ not at all
In which respect? (multiple answers possible)	◻ Professional expertise	◻ Team behaviour	
	◻ Interaction with patients	◻ Interaction with relatives	
	◻ Quality of teaching	◻ Interaction with students	
	◻ Human-personal characteristics (self-criticism, openness, patience, honesty)	◻ Being well structured	
**Others: ....**	◻ negative	◻ positive	◻ not at all
In which respect? (multiple answers possible)	◻ Professional expertise	◻ Team behaviour	
	◻ Interaction with patients	◻ Interaction with relatives	
	◻ Quality of teaching	◻ Interaction with students	
	◻ Human-personal characteristics (self-criticism, openness, patience, honesty)	◻ Being well structured	

Clinical Placement Study: Concluding questionnaire

1. The experiences during the clinical placement have increased the ‘global’ attractiveness of the chosen specialty as a personal career goal

Strongly disagree	Disagree	Neither agree nor disagree	Agree	Strongly agree
◻	◻	◻	◻	◻

2. The experiences during the clinical placement have decreased the ‘global’ attractiveness of the chosen specialty as a personal career goal

Strongly disagree	Disagree	Neither agree nor disagree	Agree	Strongly agree
◻	◻	◻	◻	◻

3. The experiences during the clinical placement have neither increased nor decreased the ‘global’ attractiveness of the chosen specialty as a personal career goal

Strongly disagree	Disagree	Neither agree nor disagree	Agree	Strongly agree
◻	◻	◻	◻	◻

4. After your experiences in the clinical placement: From your current perspective, which factors of the experienced clinical placement are more favourable and which are more unfavourable?

	Very unfavourable				Very favourable
◦ Work-life balance	◻	◻	◻	◻	◻
◦ Family friendliness	◻	◻	◻	◻	◻
◦ Patient contact	◻	◻	◻	◻	◻
◦ My own, personal enthusiasm	◻	◻	◻	◻	◻
◦ People from the specialty as role models	◻	◻	◻	◻	◻
◦ Social recognition, status of the specialty	◻	◻	◻	◻	◻
◦ Importance of the specialty for my own education	◻	◻	◻	◻	◻
◦ Importance of the specialty for my own career	◻	◻	◻	◻	◻
◦ ………………………………	◻	◻	◻	◻	◻

5. On average, how much time did you spend each day on self-study?

• During working hours	◻ < 0.5 h	◻ 0.5-1 h	◻ 1-2 h	◻ > 2 h
• During leisure time	◻ < 0.5 h	◻ 0.5-1 h	◻ 1-2 h	◻ > 2 h
